# Effects of Exercise Frequency and Intensity on Reducing Depressive Symptoms in Older Adults With Insomnia: A Pilot Randomized Controlled Trial

**DOI:** 10.3389/fphys.2022.863457

**Published:** 2022-04-05

**Authors:** Edwin C. Chin, Angus P. Yu, Chit K. Leung, Joshua D. Bernal, Whitney W. Au, Daniel Y. Fong, Calvin P. Cheng, Parco M. Siu

**Affiliations:** ^1^Division of Kinesiology, School of Public Health, The University of Hong Kong, Pokfulam, Hong Kong SAR, China; ^2^School of Nursing, The University of Hong Kong, Pokfulam, Hong Kong SAR, China; ^3^Department of Psychiatry, The University of Hong Kong, Pokfulam, Hong Kong SAR, China

**Keywords:** depression, sleep, walking, metabolic equivalent

## Abstract

**Background:**

The effects of exercise frequency and intensity on alleviating depressive symptoms in older adults with insomnia are unclear.

**Purpose:**

The purpose of this study was to investigate the influence of different exercise frequencies and intensities on prescribed aerobic-type physical activity (i.e., 75 min of vigorous-intensity exercise or 150 min of moderate-intensity exercise weekly) for reducing depressive symptoms in older adults living with insomnia, as recommended by the WHO.

**Design:**

This study is a randomized, controlled, assessor-blinded trial.

**Setting:**

This study is conducted at a single research site in Hong Kong.

**Participants:**

This study includes older adults aged 50 years or above with depressive symptoms and insomnia.

**Intervention:**

Participants were randomly assigned in a 1:1:1:1:1 ratio to the following groups: attention control (CON), moderate walking once weekly (MOD × 1/week), moderate walking thrice weekly (MOD × 3/week), vigorous walking once weekly (VIG × 1/week), and vigorous walking thrice weekly (VIG × 3/week). The total weekly exercise volumes among the walking groups were matched to the minimum recommended physical activity volume.

**Measurements:**

Depression, anxiety, self-perceived sleep quality, insomnia severity, actigraphy-assessed 7-day sleep data, 7-day sleep diary, cardiorespiratory fitness, adherence, and habitual physical activity were examined at baseline and after 12 weeks of intervention.

**Results:**

Both MOD × 3/week and VIG × 3/week groups demonstrated reduced depression (Hospital Anxiety and Depression Scale [HADS] – Depression: MOD × 3/wk: −68.6%; VIG × 3/week: −67.4%) and anxiety levels (HADS – Anxiety: MOD × 3/week: −54.3%; VIG × 3/week: −59.8%) compared with CON (both *p* < 0.01). Self-perceived sleep quality was improved in MOD × 3/week (−31.4% of the Pittsburgh Sleep Quality Index [PSQI]), VIG × 1/week (−34.1% of PSQI), and VIG × 3/week (−38.3% of PSQI), but not in MOD × 1/week, when compared with CON (*p* < 0.05). No serious adverse events were observed in this study.

**Conclusion:**

The effects of walking training on reducing depressive symptoms appeared to be dependent on exercise frequency. Our findings suggest that three sessions of walking per week at either moderate or vigorous-intensity effectively alleviate depressive symptoms in older adults with insomnia. Additional research is needed to further verify the effects of exercise frequency on depression.

**Clinical Trial Registration:**

[ClinicalTrials.gov], identifier [NCT04354922].

## Introduction

The data from the Global Burden of Disease Study 2019 showed that depressive disorder was ranked as one of the top non-communicable diseases contributing to disability-adjusted life-years (GBD 2019 Diseases and Injuries Collaborators, 2020). Depressive symptoms are highly comorbid and are associated with poor health, including cognitive decline (Gatchel et al., 2019), diabetes (Yu et al., 2015), cardiovascular disease (Correll et al., 2017; Li et al., 2019), cardiovascular mortality (Meng et al., 2020), and all-cause mortality (Meng et al., 2020). Depression is a common health problem among older adults, and the average expected prevalence of depression among old age was 31.7% (Zenebe et al., 2021). Although depression is preventable, early detection can be challenging due to the stigma of depression, especially in the Chinese population (Georg Hsu et al., 2008).

Besides being a risk factor for the onset of depression (Neckelmann et al., 2007), insomnia is also a well-recognized core symptom of depression (Nutt et al., 2008). According to the fifth edition of the Diagnostic and Statistical Manual of Mental Disorders (DSM-5) (American Psychiatric Association [APA], 2013), chronic insomnia represents a risk factor for depressive disorder and is one of the early symptoms. This is also supported by previous epidemiological studies, observing that insomnia frequently occurs before diagnosis in the majority of mood disorder cases (Ohayon and Roth, 2003). Moreover, depression is also commonly found to be comorbid in patients with insomnia or impaired sleep (Pearson et al., 2006). Insomnia is highly prevalent among the older population worldwide, with almost half of all older adults suffering from insomnia (Schubert et al., 2002). Given that insomnia is intimately associated with depression and is common among older adults, targeting older insomniac adults with depressive symptoms might be a feasible approach for the identification of early-stage depression for appropriate interventions to minimize further development of clinical depression.

Previous epidemiological evidence has indicated that increasing the level of physical activity might be an effective strategy for preventing the development of depression (Choi et al., 2019). In addition to preventing the development of depression, exercise can also serve as an effective adjunct therapy for treating depression. Moreover, exercise is relatively low cost and has a minimal risk of serious adverse effects compared with conventional treatments, such as antidepressant drugs and psychotherapy. Three meta-analyses demonstrated that exercise effectively reduced depressive symptoms compared with different types of controls, including no intervention, usual care, and placebo controls in adults diagnosed with depression (Josefsson et al., 2014; Kvam et al., 2016; Schuch et al., 2016). According to the recommendations on physical activity and sedentary behavior by the WHO, all older adults should undertake 75–150 min of vigorous-intensity [i.e., ≥6.0 metabolic equivalents of task (METs)] or 150–300 min of moderate-intensity (i.e., 3.0–5.9 METs) aerobic-type physical activity per week or an equivalent combination of both to reduce the symptoms of depression and improve sleep (Bull et al., 2020). However, there is no specific recommendation regarding the frequency prescription (i.e., sessions per week) on the WHO’s physical activity recommendation. A review from Powell et al. (2011) showed similar general health benefits of exercise at different frequencies of more than 3 days weekly, but the health benefits of exercise at frequencies less than 3 days weekly have been rarely examined. In terms of depression, a randomized controlled trial showed that either aerobic exercise 3 days weekly or 5 days weekly induced similar effects on reducing the depression score. In terms of exercise intensity, the results from a meta-analytical study demonstrated that the intensity of an endurance exercise intervention did not have a moderating effect on depression (Nebiker et al., 2018), suggesting that the effect of aerobic exercise on depression might not be intensity-dependent. Nonetheless, there are epidemiological data to show that the current physical activity recommendations performed at a lower frequency in a weekend warrior physical activity pattern (i.e., performing the recommended weekly volume of physical activity in 1–3 days weekly) may be sufficient to reduce the risk of all-cause, cardiovascular disease, and cancer mortality (O’Donovan et al., 2017). Given that time commitment is one of the most frequently reported barriers to engage in physical activity, the lower frequency weekend warrior pattern might offer flexibility and practicality to promote physical activity in the present busy lifestyle. However, there is a lack of scientific evidence on the effects of exercise frequency for alleviating depressive symptoms in older insomniac adults. Therefore, the aim of this study was to examine the effects of different frequencies and different intensities of walking training in alignment with the WHO’s physical activity recommendations for alleviating depressive symptoms in older adults living with insomnia. This study tested the hypothesis that, compared with attention control, all frequencies and intensities of aerobic waking groups reduce depressive symptoms in older adults with insomnia.

## Materials and Methods

### Study Design

This study adhered to the reporting guideline of the Consolidated Standards of Reporting Trials (CONSORT) extension for pilot or feasibility trial (Eldridge et al., 2016). This study was a pilot assessor-blinded randomized controlled trial conducted at a single research site in Hong Kong. Participants were randomly assigned to one of the five groups in 1:1:1:1:1 ratio, namely, (1) attention control (CON) group, (2) 150-min session of moderate walking once weekly (MOD × 1 day/week), (3) 50-min session of moderate walking thrice weekly (MOD × 3 days/week), (4) 75-min session of vigorous walking once weekly (VIG × 1 day/week), and (5) 25-min session of vigorous walking thrice weekly (VIG × 3 days/week). The total weekly exercise volume was matched in all walking groups according to the minimum physical activity volume recommended by WHO. All participants were instructed to maintain their normal daily physical activities. Outcome measurements were conducted before and after the 12-week intervention.

### Participants

**FIGURE 1:**
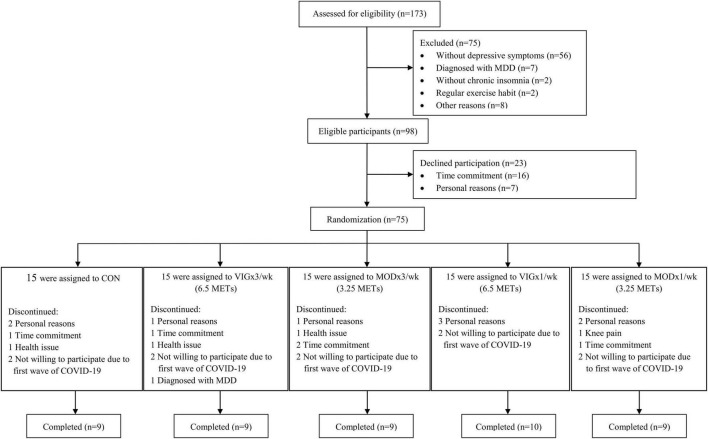
Flow diagram of this study.

This study started in January 2019 and ended in September 2021. A total of 173 potential participants were recruited from January 2019 to May 2021 through community-based promotions (Figure 1). They were invited to a face-to-face screening for depressive symptoms and chronic insomnia. All participants were provided with written and verbal information on the study protocol and on the possible risks and discomforts associated with the exercise interventions and assessments. Written informed consent was obtained prior to the start of the study. The study protocol and consent form were approved by the Institutional Review Board of the University of Hong Kong/Hospital Authority Hong Kong West Cluster (reference number: UW18-419). The study was retrospectively registered at ClinicalTrials.gov (identifier: NCT04354922) on January 13, 2020, after participant recruitment but before data analysis. All procedures were performed in this study following the Ethical Standards of the Declaration of Helsinki.

### Eligibility Criteria

Inclusion criteria include (1) borderline abnormal depression (Hospital Anxiety and Depression Scale [HADS] depression subscale ≥ 8 of 21), but not major depressive disorder; (2) insomnia symptoms present for at least 3 months according to the insomnia diagnostic criteria from the fifth edition of the DSM-5 (12); (3) individuals aged ≥50; and (4) Cantonese, Mandarin, or English speaking. Exclusion criteria include (1) contraindications to participate in physical exercise; (2) regular exercise habit in the past 3 months (>150 min of moderate-intensity exercise or 75 min of vigorous-intensity exercise weekly); (3) shift work or other commitment that can interfere with a regular sleep pattern at night; (4) individuals diagnosed with any psychiatric diseases; (5) individuals who are currently receiving non-medication treatment, such as psychotherapy or cognitive behavioral therapy for depression or insomnia; (6) abnormal cardiac electrical activity, such as myocardial infarction, arrhythmia, and irregular heartbeat, as assessed by the resting and exercise electrocardiography test; and (7) pre-existing medical or physical issues that can affect the experimental tests and exercise intervention.

### Sample Size Estimation

To the best of our knowledge, none of the previous studies have examined the effect of walking exercise once weekly on alleviating depressive symptoms among older adults. In this pilot study, we adopt a moderate effect size (interaction Cohen’s *d* of 0.5), 80% statistical power, and an α = 0.05 to estimate the sample size. A total of 45 participants (9 participants per intervention group) were required to observe the significant intervention-by-time interaction effect.

### Randomization and Masking

All eligible participants were randomly assigned to five groups in a 1:1:1:1:1 ratio. Randomization was conducted by an automated permuted block of size 5. The computer-generated randomization sequence was prepared by independent research personnel and concealed from the researcher who was responsible for participant recruitment and outcome assessments. All outcome assessors were blinded to group allocation.

### Interventions

#### Walking Interventions

Walking interventions were conducted individually using motor-driven treadmills and were supervised by certified athletics coaches or exercise physiologists (ACSM-EP). Participants in MOD × 1 day/week received one 150-min walking session weekly, and those in MOD × 3 days/week received three 50-min walking sessions weekly. Participants in VIG × 1 day/week received one 75-min session of brisk walking weekly, and those in VIG × 3 days/week received three 25-min sessions of brisk walking weekly. Participants in MOD and VIG groups walked at an exercising heart rate equivalent to 3.25 and 6.5 METs, respectively. One MET was equivalent to 3.5 ml/kg/min of oxygen consumption collected by a gaseous analysis system during the cardiopulmonary test. The exercising heart rate was continuously monitored using a heart rate monitor (M300 and H7; Polar Electro Oy, Finland) and recorded during the walking sessions. Participants were instructed to maintain their exercising heart rate to within ±10 beats per minute (bpm) of their prescribed walking heart rate. Participants in the MOD × 1 day/week and VIG × 1 day/week groups were allowed to take one or two breaks for 5–10 min during each training session. All intensities and volumes of the walking interventions were adhered to the WHO’s physical activity recommendations (Bull et al., 2020).

#### Control Group

Participants in the CON group received one 75-min session of stretching exercises weekly. The contact duration of the stretching sessions was designed to match that of the VIG groups. The stretching sessions were supervised by certified athletics coaches.

### Outcome Measures

Trained outcome assessors were blinded to the participant’s group allocation. All questionnaire-based outcomes were conducted by an assessor-led semi-structured interview.

#### Depressive and Anxiety Symptoms

The Chinese-Cantonese version of HADS was used to evaluate psychological distress in terms of depressive and anxiety symptoms. This questionnaire has been validated in Hong Kong Chinese adults (Leung et al., 1999) with excellent reliability (Cronbach’s alpha: overall scale = 0.86, depression subscale = 0.82, anxiety subscale = 0.77). It has been demonstrated to have a significant correlation with the Hamilton Rating of Depression (*r* = 0.67, *p* < 0.001) and Anxiety (*r* = 0.63, *p* < 0.001), which confirms its validity. This 14-item instrument has seven items in the depression subscale and seven items in the anxiety subscale. Each subscale has an overall score ranging from 0 to 21, with a higher score indicating a higher level of depression or anxiety.

#### Symptom Severity of Depression and Anxiety

The Patient Health Questionnaire-9 (PHQ-9) and Generalized Anxiety Disorder-7 (GAD-7) were used to supplement the assessments of the occurrence and severity of depression and anxiety symptoms. PHQ-9 is a 9-item instrument derived from the criteria of DSM-5 and measures the heterogeneous spectrum of symptoms of major depressive disorder (American Psychiatric Association [APA], 2013). This 9-item questionnaire asks how often the participants were bothered by their depressive symptoms in the past 2 weeks. The response options are “not at all” (score 0), “several days” (score 1), “more than half the days” (score 2), and “nearly every day” (score 3). The overall scores are used to categorize the severity of the depressive symptoms according to the suggested cutoff scores (0–4 = minimal depression, 5–9 = mild depression, 10–14 = moderate depression, 15–19 = moderately severe depression, and ≥20 = severe depression). The Chinese version of PHQ-9 has been validated in Hong Kong adults with excellent reliability (Cronbach’s alpha = 0.82) (Yu et al., 2012). GAD-7 is a 7-item questionnaire modified from the diagnostic criteria of DSM-5 and is designed to screen GAD and evaluate its severity (Spitzer et al., 2006). Each item describes a symptom of anxiety disorder and the frequency of the symptom over the past 2 weeks. The response option and scoring method are similar to PHQ-9. The overall scores in GAD-7 are used to evaluate the severity of anxiety based on the suggested cutoff scores (5–9 = mild anxiety, 10–14 = moderate anxiety, and 15 = severe anxiety). The Chinese version of GAD-7 has been validated in general hospital outpatients (Cronbach’s alpha = 0.898) (He et al., 2010).

#### Subjective Sleep Parameters

The Chinese version of the Pittsburgh Sleep Quality Index (PSQI) was used to measure self-perceived sleep quality. It consists of 19 items that measure seven sleep components, including sleep quality, sleep latency, sleep duration, habitual sleep efficiency, sleep disturbances, sleeping medication use, and daytime dysfunction. Each component is scored on a scale of 0–3, with 0 indicating no sleep problems and 3 indicating sleep impairments. The overall global score of the seven components ranges from 0 to 21. The Chinese version of PSQI has been demonstrated to have satisfactory test-retest reliability of 0.85 in insomniac and general populations (Tsai et al., 2005). The Insomnia Severity Index (ISI) questionnaire was used to measure the perceived insomnia severity. This 7-item instrument includes questions on sleep-onset and sleep maintenance difficulties, satisfaction with the current sleep pattern, interference with daily functioning, noticeability of impairments due to sleep problems, degree of distress, and concerns due to sleep problems. Each item is rated on a three-point Likert-type scale with a higher score indicating more perceived insomnia severity. The Chinese version of ISI has been shown to have an acceptable content validity index of 0.94 and high internal consistency (Cronbach’s alpha = 0.81) in Hong Kong Chinese older adults (Yu, 2010).

#### Seven-Day Actigraphic Measure of Sleep and Habitual Physical Activity

A 3-axis accelerometer (wGT3X-BT, Actigraph) was used to measure and record movement during sleep. Participants were instructed to wear the accelerometer on the non-dominant wrist during sleep for 7 days. The movement data during sleep were used to determine sleep efficiency, wake time after sleep onset, the number of awakenings per night, sleep onset latency, total sleep time, and average wake time per awakening. The ActiLife version 6.11.7 software was used to analyze the sleep data extracted from the accelerometer. As one of the major confounding factors in this study, habitual physical activity level was assessed before and after the intervention. The ActiGraph accelerometer (wGT3X-BT, ActiGraph, United States), which has been previously validated (Plasqui and Westerterp, 2007), was used to measure the habitual physical activity. Participants were instructed to wear the ActiGraph watch on the non-dominant arm for 7 days. The physical activity data were processed using ActiLife version 6.11.7 software to measure the time spent in sedentary activities, light-intensity activities, moderate-intensity activities, vigorous-intensity activities, and very vigorous-intensity activities.

#### Seven-Day Sleep Diary

Participants were instructed to record their sleep patterns in a sleep log each morning for 7 days at all assessment time points. Participants were asked to record bedtime, sleep rising time, wake time after sleep onset, total sleep time, number of awakenings, and sleep onset latency. Sleep efficiency was estimated by (total sleep time/total time in bed) × 100%. Average awakening time was estimated by (wake time after sleep onset/number of awakenings). Although the sleep diary is a participative measure, it has been shown to be a reliable instrument for collecting data on sleep/wake patterns. An acceptable percentage agreement between the sleep diary data and polysomnographic data (kappa = 0.87) has been previously demonstrated (Rogers et al., 1993). The dosage and frequency of sleep aid medications or supplements, such as narcotics, antihistamines (diphenhydramine), benzodiazepines (e.g., flurazepam, quazepam, triazolam, estazolam, temazepam, clonazepam, lorazepam, and alprazolam), non-benzodiazepine, benzodiazepine receptor agonists (e.g., zolpidem, zaleplon, and eszopiclone), or melatonin, were also recorded in the 7-day sleep diary.

#### Cardiorespiratory Fitness

A progressive cardiopulmonary test was conducted on a calibrated motor-driven treadmill (T150 DE LC MED, COSMED). Gaseous analysis system (Quark CPET, COSMED) and 12-lead electrocardiogram (Quark T12×, COSMED) was used to continuously measure and record the VO_2_ and heart rate, respectively. The rate of perceived exertion (RPE) on a scale of 6–20 was measured every 3 min during this test. The modified Bruce treadmill protocol was used, in which the intensity was continuously increased every 3 min until the maximal oxygen consumption (VO_2*max*_) was reached. The VO_2*max*_ determination criteria were determined by the plateau of VO_2_ with increasing intensity, or meeting at least two of the following criteria: (1) respiratory exchange ratio ≥ 1.10, (2) within 10 bpm of the age-predicted maximal heart rate (220-age), or (3) RPE ≥ 18 (Hanson et al., 2016).

### Statistical Analysis

A generalized estimating equations (GEEs) model with baseline as a covariate was employed to assess the intervention effects on the outcomes after accounting for the extra-covariance among the repeated measurements. The GEE analysis was conducted using the R package “geeM.” The *post hoc* comparison among groups was performed by a closed test procedure using the R package “multcomp.” One-way ANOVA was used to examine between-group differences in adherence assessed at post-intervention. The Pearson product-moment correlation coefficient test was used to examine the association between depressive symptoms and cardiorespiratory fitness changes. All statistical analyses were performed by R (version 3.6.1). Statistical significance was set at a *p*-value < 0.05, and the Cohen’s *d* was calculated to estimate the effect size of significant between-group findings. Data were expressed as mean ± SD.

## Results

### Baseline Characteristics of Participants

A total of 173 participants were screened for depressive symptoms and chronic insomnia by semi structured interviews (Figure 1) from January 2019. Overall, 75 participants were randomly assigned to CON (*n* = 15), MOD × 1 day/week (*n* = 15), MOD × 3 days/week (*n* = 15), VIG × 1 day/week (*n* = 15), and VIG × 3 days/week (*n* = 15) within 1 month after the baseline assessment. Notably, 46 participants (age: 63.3 ± 5.5 years, 15% male) were completed the intervention and included in the analysis (CON: *n* = 9; MOD × 1/week: *n* = 9; MOD × 3/week: *n* = 9; VIG × 1/week: *n* = 10; VIG × 3/week: *n* = 9). The baseline characteristics of participants are summarized in Table 1. One subject in the MOD × 1/week group reported knee pain after training (Figure 1).

**TABLE 1:** Baseline characteristics of participants.

	Participants, mean ± SD				
	CON (*n* = 9)	MOD × 1/week (*n* = 9)	MOD × 3/week (*n* = 9)	VIG × 1/week (*n* = 10)	VIG × 3/week (*n* = 9)
Male, *n* (%)	1.0 (11.1)	2.0 (22.2)	2.0 (22.2)	3.0 (33.3)	0.0 (0.0)
Age	63.8 ± 6.0	65.9 ± 7.0	63.7 ± 4.7	61.5 ± 6.1	61.7 ± 2.7
HADS-depression score	11.1 ± 3.1	9.9 ± 1.5	9.6 ± 1.9	10.1 ± 1.4	10.2 ± 2.5
Insomnia duration (months)	85.3 ± 62.9	81.7 ± 92.4	86.7 ± 107.9	76.3 ± 74.9	63.4 ± 69.3
**History of psychiatric disease**					
Major depressive disorder, *n* (%)	0.0 (0.0)	0.0 (0.0)	0.0 (0.0)	0.0 (0.0)	1.0 (11.1)
Anxiety disorder, *n* (%)	0.0 (0.0)	0.0 (0.0)	0.0 (0.0)	0.0 (0.0)	0.0 (0.0)
Sleep disorders, *n* (%)	0.0 (0.0)	1.0 (11.1)	0.0 (0.0)	1.0 (10.0)	1.0 (11.1)
Hypnotic medication usage, *n* (%)	0.0 (0.0)	1.0 (11.1)	0.0 (0.0)	1.0 (10.0)	1.0 (11.1)

### Depression and Anxiety

**FIGURE 2:**
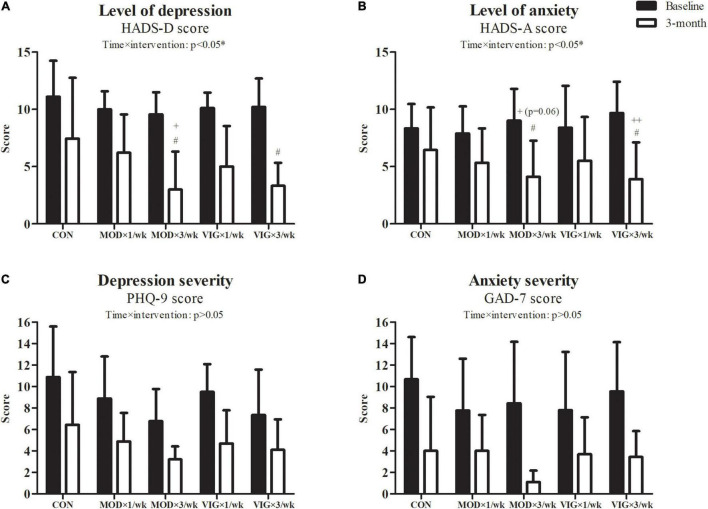
Level of depression **(A)**, level of anxiety **(B)**, depression severity **(C)**, and anxiety severity **(D)** were examined at baseline and after the 12-week intervention. Control (CON; attention CON group) = one session of 75 min stretching exercise weekly, VIG × 3/week = three sessions of 25 min vigorous-intensity [6.5 metabolic equivalents of task (METs)] walking exercise weekly, MOD × 3/week = three sessions of 50 min moderate-intensity (3.25 METs) walking exercise weekly, VIG × 1/week = one session of 75 min vigorous-intensity (6.5 METs) walking exercise weekly, MOD × 1/week = one session of 150 min vigorous-intensity (3.25 METs) walking exercise weekly. ^#^Significantly different from CON at the same time point. ^+^Significantly different from MOD × 1/week at the same time point. ^++^Significantly different from VIG × 1/week at the same time point.

The results of the outcome measures on depression and anxiety are shown in Figure 2. Our GEE analyses revealed a group-by-time interaction effect in HADS-depression (interaction effect, *p* = 0.02) and HADS-anxiety (interaction effect, *p* = 0.02). According to the *post hoc* analyses, HADS-depression and HADS-anxiety were lower in MOD × 3 days/week and VIG × 3 days/week groups after 12 weeks of walking training compared to CON (HADS-depression: MOD × 3 days/week–CON, Cohen’s *d* = 1.57, *p* = 0.004; VIG × 3 days/week–CON, Cohen’s *d* = −2.21, *p* = 0.0003) (HADS-anxiety: MOD × 3 days/week–CON, Cohen’s *d* = 1.02, *p* = 0.01; VIG × 3 days/week–CON, Cohen’s *d* = −1.31, *p* = 0.001). Furthermore, HADS-depression was lower in MOD × 3 days/week than in MOD × 1 day/week (MOD × 3 days/week–MOD × 1 day/week, Cohen’s *d* = −0.97, *p* = 0.01), whereas it only tended to be lower in the VIG × 3 days/week than in VIG × 1 day/week (VIG × 3 days/week–VIG × 1 day/week, Cohen’s *d* = −1.16, *p* = 0.07) after 12 weeks of walking training. HADS-anxiety improved with a higher frequency of VIG training (VIG × 3 days/week–VIG × 1 day/week, Cohen’s *d* = −1.16, *p* = 0.01), whereas it only showed a trend of improvement with a higher frequency of MOD training (MOD × 3 days/week–MOD × 1 day/week, Cohen’s *d* = −0.70, *p* = 0.06). No significant group-by-time interaction effects were found in the severity of depression and anxiety measured by PHQ-9 and GAD-7, respectively.

### Self-Reported Sleep Parameters

**FIGURE 3:**
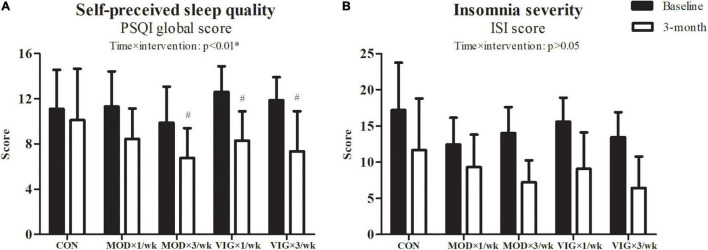
Self-perceived sleep quality **(A)** and insomnia severity **(B)** were examined at baseline and after the 12-week intervention. CON (attention CON group) = one session of 75 min stretching exercise weekly, VIG × 3/week = three sessions of 25 min vigorous-intensity (6.5 METs) walking exercise weekly, MOD × 3/week = three sessions of 50 min moderate-intensity (3.25 METs) walking exercise weekly, VIG × 1/week = one session of 75 min vigorous-intensity (6.5 METs) walking exercise weekly, MOD × 1/week = one session of 150 min vigorous-intensity (3.25 METs) walking exercise weekly. ^#^Significantly different from CON at the same time point.

Interaction effects were also observed in the self-perceived sleep quality (*p* = 0.001) (Figure 3). The *post hoc* comparisons showed that both groups with three sessions weekly had improved self-perceived sleep quality compared with the CON group (MOD × 3 days/week–CON, Cohen’s *d* = 0.82, *p* = 0.02; VIG × 3 days/week–CON, Cohen’s *d* = −1.32, *p* = 0.001), whereas only the once-weekly vigorous-intensity walking showed improved self-perceived sleep quality (VIG × 1 day/week–CON, Cohen’s *d* = 1.52, *p* = 0.002).

### Actigraphy-Assessed Sleep Parameters and Sleep Diary

The results of sleep parameters assessed by actigraphy and sleep diary are presented in Table 2. There were five missing actigraphic data (CON: *n* = 2, MOD × 1 day/week: *n* = 2, MOD × 3 days/week: *n* = 1) in the analysis due to participants feeling discomfort in wearing the ActiGraph watch during sleep (CON: *n* = 1; MOD × 1 day/week: *n* = 1; MOD × 3 days/week: *n* = 1), skin allergy caused by the wristband (MOD × 1 day/week: *n* = 1), and file corruption during data extraction by the ActiLife software (CON: *n* = 1). Our GEE analyses revealed that there were no significant interaction effects in all the sleep parameters measured by actigraphy and sleep diary.

**TABLE 2:** Summary of the objective sleep data assessed by actigraphy and the subjective sleep data measured by 7-day sleep diary.

	Baseline	3-month	Interaction effect	Group effect	Time effect
**Actigraph-assessed sleep efficiency (%)**					
CON (*n* = 7)	84.3 ± 4.8	84.8 ± 3.3			
MOD × 1 day/week (*n* = 7)	79.6 ± 8.8	81.1 ± 9.5			
MOD × 3 days/week (*n* = 8)	83.7 ± 7.0	85.5 ± 4.5	*p* = 0.797	*p* = 0.936	*p* = 0.419
VIG × 1 day/week	85.2 ± 8.2	85.5 ± 7.9			
VIG × 3 days/week	85.1 ± 4.6	87.8 ± 2.6			
**Actigraph-assessed wake time after sleep onset (min)**					
CON (*n* = 7)	76.6 ± 41.0	72.3 ± 19.8			
MOD × 1 day/week (*n* = 7)	85.0 ± 21.6	84.7 ± 37.0			
MOD × 3 days/week (*n* = 8)	68.4 ± 28.6	65.5 ± 18.8	*p* = 0.670	*p* = 0.468	*p* = 0.458
VIG × 1 day/week	61.1 ± 31.9	61.4 ± 38.8			
VIG × 3 days/week	56.2 ± 20.6	50.4 ± 13.7			
**Actigraph-assessed number of awakenings (*n*)**					
CON (*n* = 7)	21.0 ± 6.4	21.8 ± 5.1			
MOD × 1 day/week (*n* = 7)	21.8 ± 2.3	18.0 ± 3.2			
MOD × 3 days/week (*n* = 8)	20.4 ± 6.9	20.6 ± 4.1	*p* = 0.149	*p* = 0.036	*p* = 0.117
VIG × 1 day/week	15.9 ± 4.3	16.5 ± 5.4			
VIG × 3 days/week	15.4 ± 5.3	15.2 ± 4.2			
**Actigraph-assessed average awaken time (min)**					
CON (*n* = 7)	3.6 ± 1.0	3.4 ± 1.0			
MOD × 1 day/week (*n* = 7)	4.2 ± 1.2	5.0 ± 2.5			
MOD × 3 days/week (n = 8)	3.6 ± 2.0	3.3 ± 0.9	*p* = 0.310	*p* = 0.335	*p* = 0.555
VIG × 1 day/week	3.7 ± 1.7	3.6 ± 1.2			
VIG × 3 days/week	3.9 ± 1.4	3.3 ± 0.7			
**Actigraph-assessed sleep onset latency (min)**					
CON (*n* = 7)	4.1 ± 1.6	4.5 ± 2.6			
MOD × 1 day/week (*n* = 7)	7.3 ± 10.3	3.9 ± 3.3			
MOD × 3 days/week (*n* = 8)	5.2 ± 3.7	4.5 ± 6.6	*p* = 0.800	*p* = 0.708	*p* = 0.510
VIG × 1 day/week	4.1 ± 3.3	5.4 ± 3.5			
VIG × 3 days/week	8.3 ± 4.9	4.2 ± 2.1			
**Actigraph-assessed total sleep time (min)**					
CON (*n* = 7)	411.2 ±	420.0 ± 106.9			
	60.6				
MOD × 1 day/week (*n* = 7)	378.9 ±	415.4 ± 61.5			
	69.3				
MOD × 3 days/week (*n* = 8)	376.1 ±	435.9 ± 126.9	*p* = 0.121	*p* = 0.045	*p* = 0.614
	67.4				
VIG × 1 day/week	386.7 ±	468.2 ± 98.8			
	85.6				
VIG × 3 days/week	362.7 ±	467.3 ± 79.8			
	39.0				
**Sleep efficiency assessed by 7-day sleep diary (%)**					
CON	77.2 ± 7.0	76.2 ± 9.9			
MOD × 1 day/week	79.9 ± 9.8	85.7 ± 9.6			
MOD × 3 days/week	81.1 ± 14	88.6 ± 4.7	*p* = 0.990	*p* = 0.561	*p* = 0.336
VIG × 1 day/week	82.1 ± 8.5	81.1 ± 10.2			
VIG × 3 days/week	86.0 ± 7.9	88.8 ± 6.2			
**Wake time after sleep onset assessed by 7-day sleep diary (min)**					
CON	58.3 ± 35.3	68.0 ± 49.5			
MOD × 1 day/week	71.7 ± 49.6	49.0 ± 49.2			
MOD × 3 days/week	57.3 ± 59.9	19.6 ± 16.6	*p* = 0.962	*p* = 0.610	*p* = 0.481
VIG × 1 day/week	51.5 ± 42.2	58.4 ± 40.4			
VIG × 3 days/week	30.5 ± 30.2	23.4 ± 24.3			
**Number of awakenings assessed by 7-day sleep diary (n)**					
CON	2.3 ± 1.1	2.5 ± 0.7			
MOD × 1 day/week	2.4 ± 1.0	2.8 ± 1.5			
MOD × 3 days/week	1.9 ± 1.3	2.5 ± 1.8	*p* = 0.171	*p* = 0.282	*p* = 0.111
VIG × 1 day/week	2.4 ± 0.9	2.3 ± 1.0			
VIG × 3 days/week	1.7 ± 0.8	1.4 ± 0.7			
**Average awakening times assessed by 7-day sleep diary (min)**					
CON	38.6 ± 33.0	29.8 ± 24.7			
MOD × 1 day/week	33.2 ± 24.1	20.0 ± 18.7			
MOD × 3 days/week	36.4 ± 30.4	11.2 ± 9.5	*p* = 0.179	*p* = 0.118	*p* = 0.017
VIG × 1 day/week	26.1 ± 23.6	29.2 ± 28.1			
VIG × 3 days/week	19.3 ± 14.8	34.5 ± 68.2			
**Sleep onset latency assessed by 7-day sleep diary (min)**					
CON	55.4 ± 42.3	57.9 ± 40.7			
MOD × 1 day/week	34.2 ± 14.1	22.9 ± 9.4			
MOD × 3 days/week	34.4 ± 21.3	36.2 ± 27.5	*p* = 0.984	*p* = 0.771	*p* = 0.875
VIG × 1 day/week	30.9 ± 16.2	34.2 ± 28.8			
VIG × 3 days/week	32.1 ± 13.1	27.7 ± 19.5			
**Total sleep time assessed by 7-day sleep diary (min)**					
CON	386.0 ±	392.7 ± 40.0			
	71.6				
MOD × 1 day/week	398.0 ±	408.0 ± 46.7			
	34.5				
MOD × 3 days/week	368.0 ±	420.7 ± 45.1	*p* = 0.496	*p* = 0.393	*p* = 0.332
	57.9				
VIG × 1 day/week	360.0 ±	374.4 ± 46.5			
	42.5				
VIG × 3 days/week	370.7 ±	392.7 ± 45.2			
	27.1				
**Hypnotic medication usage recorded by 7-day sleep diary (LRD units/week)**					
CON					
MOD × 1 day/week	2.0 (*n* = 1)	3.5 (*n* = 1)			
MOD × 3 days/week	4.0 (*n* = 1)	2.0 (*n* = 1)	N/A	N/A	N/A
VIG × 1 day/week					
VIG × 3 days/week	2.0 (*n* = 1)	2.0 (*n* = 1)			

### Cardiorespiratory Fitness, Adherence to Intervention, Walking Heart Rate, Speed, and Inclination

**FIGURE 4:**
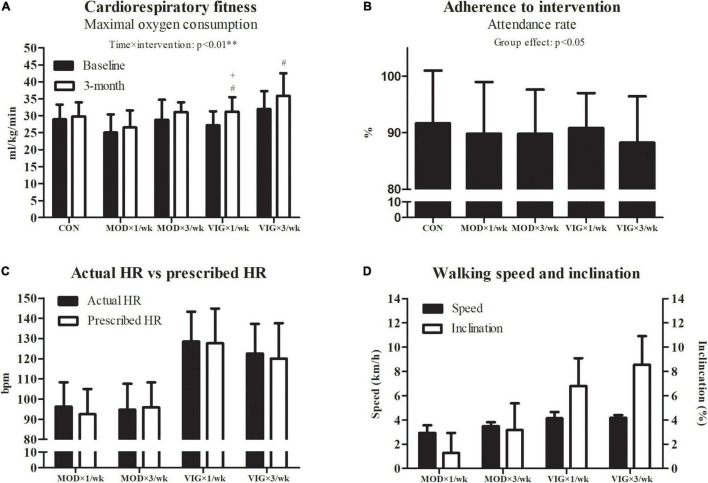
Cardiorespiratory fitness **(A)**, adherence **(B)**, actual walking heart rate vs. prescribed heart rate **(C)**, and walking speed and inclination **(D)**. CON (attention CON group) = one session of 75 min stretching exercise weekly, VIG × 3/week = three sessions of 25 min vigorous-intensity (6.5 METs) walking exercise weekly, MOD × 3/week = three sessions of 50 min moderate-intensity (3.25 METs) walking exercise weekly, VIG × 1/week = one session of 75 min vigorous-intensity (6.5 METs) walking exercise weekly, MOD × 1/week = one session of 150 min vigorous-intensity (3.25 METs) walking exercise weekly. ^#^Significantly different from CON at the same time point.^ +^Significantly different from MOD × 1/week at the same time point.

A significant interaction effect was detected in the cardiorespiratory fitness (*p* = 0.001) (Figure 4). The *post hoc* analyses showed that all VIG groups, but not MOD groups, had improved cardiorespiratory fitness compared with CON (VIG × 1 day/week–CON, Cohen’s *d* = 0.75, *p* = 0.005; VIG × 3 days/week–CON, Cohen’s *d* = 0.45, *p* = 0.001). The improvements in cardiorespiratory fitness in VIG × 1 day/week were greater than that in MOD × 1 day/week (VIG × 1 day/week–MOD × 1 day/week, Cohen’s *d* = 0.66, *p* = 0.008). Furthermore, the Pearson product-moment correlation coefficient test revealed no significant correlation between the change in cardiorespiratory fitness and HADS-depression (*r* = −0.18, *p* = 0.24). No significant differences were found in the adherence across all groups. The mean and SD of the walking heart rate, speed, and inclination are presented in Figures 4C,D, respectively.

### Habitual Physical Activities and Body Weight

The data on habitual physical activities and body weight are presented in Table 3. No significant interaction effects were detected across all intensity levels of habitual physical activities and body weight.

**TABLE 3:** Summary of the habitual physical activities and body weight.

	Baseline	3-months	Interaction effect	Group effect	Time effect
**Sedentary activities (min)**					
CON (*n* = 7)	778.4 ± 168.5	844.2 ± 141.6			
MOD× 1day/week (*n* = 7)	836.2± 69.3	846.0 ± 136.4			
MOD× 3days/week (*n* = 8)	800.8± 144.0	830.5 ± 145.7	*p* = 0.553	*p* = 0.265	*p* = 0.886
VIG× 1day/week	736.2± 135.9	764.2 ± 252.0			
VIG× 3days/week	685.1± 130.5	740.7 ± 67.8			
**Light-intensity activities (min)**					
CON (*n* = 7)	420.0± 106.9	366.0 ± 72.6			
MOD× 1day/week (*n* = 7)	415.4± 61.5	409.8 ± 121.1			
MOD× 3days/week (*n* = 8)	435.9± 126.9	396.9 ± 122.8	*p* = 0.973	*p* = 0.535	*p* = 0.716
VIG× 1day/week	468.2± 98.8	439.7 ± 186.3			
VIG× 3days/week	467.3± 79.8	478.0 ± 99.4			
**Moderate-intensity activities (min)**					
CON (*n* = 7)	177.9± 101.0	155.5 ± 88.1			
MOD× 1day/week (*n* = 7)	110.0 ± 40.9	105.3 ± 39.9			
MOD× 3days/ week (*n* = 8)	148.3 ± 57.0	149.9 ± 59.9	*p* = 0.707	*p* = 0.456	*p* = 0.970
VIG× 1day/week	182.8 ± 77.9	167.0 ± 108.6			
VIG× 3days/week	164.9 ± 66.1	164.7 ± 65.2			
**Vigorous-intensity activities (min)**					
CON (*n* = 7)	0.0 ± 0.0	0.0 ± 0.0			
MOD× 1day/week (n = 7)	0.0 ± 0.0	0.0 ± 0.0			
MOD× 3days/week (*n* = 8)	0.0 ± 0.0	0.0 ± 0.0	N/A	N/A	N/A
VIG× 1day/week	0.0 ± 0.0	0.0 ± 0.0			
VIG× 3days/week	0.0 ± 0.0	0.0 ± 0.0			
**Very vigorous-intensity activities (min)**					
CON (*n* = 7)	0.0 ± 0.0	0.0 ± 0.0			
MOD× 1day/week (*n* = 7)	0.0 ± 0.0	0.0 ± 0.0			
MOD× 3days/week (*n* = 8)	0.0 ± 0.0	0.0 ± 0.0	N/A	N/A	N/A
VIG× 1day/week	0.0 ± 0.0	0.0 ± 0.0			
VIG× 3days/week	0.0 ± 0.0	0.0 ± 0.0			
Body weight (kg)					
CON	54.5 ± 10.1	53.1 ± 10.6			
MOD× 1day/week	61.2 ± 10.0	61.7 ± 10.2			
MOD× 3days/week	60.0 ± 11.8	59.2 ± 11.4	*p* = 0.231	*p* = 0.238	*p* = 0.112
VIG× 1day/week	56.3 ± 5.0	56.3 ± 5.8			
VIG× 3days/week	54.8 ± 8.5	54.6 ± 8.2			

## Discussion

This study examined the effects of exercise frequency and intensity on alleviating depressive symptoms in older adults with chronic insomnia. The main findings provide preliminary evidence on the influence of exercise frequency and intensity in several aspects. First, only the groups performing three walking sessions weekly had reduced levels of depression and anxiety. Second, exercise intensity did not appear to result in differential training effects on decreasing depressive symptoms in both once- and thrice-weekly walking groups. Third, once- and thrice-weekly vigorous walking improved subjective sleep quality. Collectively, our results suggest that the frequency of exercise might be an important factor in modulating mood and sleep in older adults living with depressive symptoms and insomnia.

At present, the WHO guidelines recommend that older adults should perform balance and muscle-strengthening activities for 3 or more days per week (Bull et al., 2020). However, specific recommendations for the frequency of aerobic-type physical activities are lacking. By matching the energy expenditure of the interventions according to the 1995 physical activity recommendations proposed by the Centers for Disease Control and Prevention of the United States and the American College of Sports Medicine, Dunn et al. (2005) demonstrated that the frequency of exercise (i.e., 3 days vs. 5 days weekly) did not alter the effects of training on reducing depressive symptoms (Pate et al., 1995). This finding is inconsistent with our results, in which the effects of training for 3 days weekly were superior to training for 1 day weekly. This discrepancy could be explained by the different study designs and the comparison of exercise frequency. In this study, no significant effects on depressive symptoms were observed in participants performing the recommended exercise volume in just 1 day per week. The effect of exercise on depressive symptoms might be diminished when the frequency is less than 3 days weekly regardless of the exercise intensity. Based on our preliminary results, a large-scale randomized controlled trial is warranted to validate this speculated minimum exercise frequency threshold for reducing depressive symptoms. The minimum frequency threshold will be critical for advising the specific exercise pattern in physical activity interventions for reducing depressive symptoms in older adults suffering from insomnia.

In terms of the effect of exercise intensity on depressive symptoms, this study found that moderate-intensity walking had similar effects to vigorous-intensity walking on alleviating depressive symptoms. This finding is consistent with a meta-analysis, showing that exercise intensity might not be an important factor modulating the effects of aerobic exercise training on depression (Nebiker et al., 2018). In our study, we used the absolute intensity value (i.e., number of METs), whereas most of the studies in the meta-analysis used the percentage of maximal heart rate or maximal oxygen consumption, which are relative intensity references. Although our study found that adopting the WHO-recommended physical activity intensities in three sessions per week over the 12-week walking program could alleviate depressive symptoms, training at a certain level of METs for a prolonged period may decrease the rate of improvement of cardiorespiratory fitness due to progressive adaptation and plateau effects of training. Further investigations with a prolonged study period might be needed to monitor the adaptation of cardiorespiratory fitness and to progressively adjust the training intensity to avoid plateau effects of training when using METs to prescribe exercise intensity. In addition, our results showed that improvement of cardiorespiratory fitness was not associated with the reduction of depressive symptoms. This observation was conflicted with a previous meta-analysis, indicating that cardiorespiratory fitness is inversely correlated with depressive symptom severity (Papasavvas et al., 2016). Although the previous studies have shown that higher intensity of aerobic exercise exhibits a greater improvement in cardiorespiratory fitness (Gormley et al., 2008), further investigations are needed to delineate the relationships among exercise intensity, exercise frequency, cardiorespiratory fitness, and depression.

Anxiety symptoms are commonly comorbid with depression (Gorman, 1996). In this study, 74% of our participants had anxiety symptoms (i.e., HADS-Anxiety score ≥ 8). Our results revealed that the effects of walking training on alleviating anxiety and depressive levels were homogeneous across all walking intervention groups. Nonetheless, our findings were in conflict with a previous large-scale cross-sectional study, demonstrating that exercise performed at least one to two times per week was associated with a lower risk of anxiety symptoms in adults with clinical anxiety (Hallgren et al., 2020), and our findings were in conflict with a meta-analysis, finding that high-intensity exercise regimens were more effective than low-intensity regimens in the general population (Aylett et al., 2018). However, there are insufficient studies that have specifically investigated the influence of exercise frequency and intensity on alleviating anxiety levels among people with depressive symptoms. Therefore, our preliminary results further extend our understanding that the beneficial adaptation due to different exercise frequencies and intensities on anxiety level might be associated with depressive level in older adults with depressive symptoms. Further investigations are needed to verify the role of exercise frequency and intensity in reducing anxiety levels in patients with clinical depression and/or anxiety.

Our results also showed that adopting the WHO-recommended physical activity level over 3 days per week could improve the PSQI global score in older adults with chronic insomnia. This finding is in line with a previous epidemiological study that found compliance with the WHO physical activity recommendations resulted in a lower risk of sleep problems (Vancampfort et al., 2018). A previous epidemiological study also suggested that the favorable effects of physical activity on depressive symptoms might be mediated through improving subjective sleep quality (Kaseva et al., 2019). Intriguingly, our results showed that both subjective sleep quality and depressive symptoms were improved after 12 weeks of thrice-weekly moderate or vigorous walking, whereas once-weekly walking sessions only enhanced the quality of sleep at a vigorous intensity. Although the mediation effect of sleep on depressive symptoms might be attenuated when the exercise frequency was reduced to once weekly, our results showed that even weekly vigorous-intensity walking can improve the perceived sleep quality when compared with weekly moderate-intensity walking. We attempted to examine the favorable effects on sleep through different objective sleep parameters from actigraphy-assessed sleep data and sleep diary logs. However, our results were not in line with a previous study that found one session of moderate-intensity aerobic exercise elicited better improvement of polysomnographic sleep measures (i.e., sleep onset latency, total sleep time, and total wake time) and sleep log (i.e., sleep onset latency and total sleep time) when compared with vigorous-intensity aerobic exercise (Passos et al., 2010). Unlike our actigraphy data collected from the usual sleep environment, an unfamiliar laboratory environment and planned sleep schedules during polysomnographic measurements might lead to sleep perturbations (Ghegan et al., 2006). This might account for the discrepancy in the objective sleep outcomes between the current study and the previous study.

Several limitations of this study need to be noted. First, our walking exercise volume only benchmarked the lowest physical activity threshold, as recommended by the WHO. The recommended aerobic-type physical activity volume for adults and older adults ranges from 150 to 300 min of moderate-intensity aerobic exercise and 75 to 150 min of vigorous-intensity aerobic exercise weekly (Bull et al., 2020). The influence of walking frequency at the highest volumes (i.e., 300 min of moderate-intensity exercise and 150 min of vigorous-intensity exercise) remains unknown. Second, all participants were trained individually, and all walking sessions were conducted on motor-driven treadmills in a laboratory environment. Although our study design eliminated the effect of group training on mood and attempted to control the training environment (e.g., temperature, humidity, and noise), the practical application of the present findings in a field setting needs to be verified in the future studies. Third, our results are specific for older adults with chronic insomnia and subclinical, but considerable, depressive symptoms. As we purposely excluded people with major depressive disorder, our findings might not be generalizable to the entire population with depression. Fourth, dietary intake may influence the management of depressive symptoms. Although our results showed no significant change in body weight after 12 weeks of the intervention, monitoring of dietary intake was lacking in this pilot study. A standardized diet across the intervention periods and a detailed dietary intake record should be implemented in future studies. Last, subjective sleep quality was associated with body composition (Jurado-Fasoli et al., 2018). Future studies should separately analyze the population with the different cutoff values of body composition parameters, such as obesity categorized by body mass index, central obesity defined by waist circumference, or osteoporosis classified by bone density levels.

In conclusion, among older adults with depressive symptoms and chronic insomnia, the effects of walking training on alleviating depressive symptoms appeared to be dependent on exercise frequency. This study demonstrated that three walking sessions weekly at either moderate or vigorous-intensity according to the physical activity recommendations by the WHO are an effective approach for managing depressive symptoms in older adults living with insomnia.

## Data Availability Statement

The raw data supporting the conclusions of this article will be made available by the authors, without undue reservation.

## Ethics Statement

The studies involving human participants were reviewed and approved by Institutional Review Board of the University of Hong Kong/Hospital Authority Hong Kong West Cluster (reference number: UW18-419). The patients/participants provided their written informed consent to participate in this study.

## Author Contributions

EC and PS designed the study and wrote and edited the manuscript. EC, AY, CL, JB, and WA performed the experiments. EC, AY, DF, CC, and PS analyzed and interpreted the data. All authors contributed to the article and approved the submitted version.

## Conflict of Interest

The authors declare that the research was conducted in the absence of any commercial or financial relationships that could be construed as a potential conflict of interest.

## Publisher’s Note

All claims expressed in this article are solely those of the authors and do not necessarily represent those of their affiliated organizations, or those of the publisher, the editors and the reviewers. Any product that may be evaluated in this article, or claim that may be made by its manufacturer, is not guaranteed or endorsed by the publisher.

## References

[B1] American Psychiatric Association [APA] (2013). *Diagnostic and Statistical Manual of Mental Disorders*, 5th Edn. Washington, DC: American Psychiatric Association.

[B2] Aylett E. Small N. Bower P. (2018). Exercise in the treatment of clinical anxiety in general practice - a systematic review and meta-analysis. *BMC Health Serv. Res.* 18:559. 10.1186/s12913-018-3313-5 30012142 PMC6048763

[B3] Bull F. C. Al-Ansari S. S. Biddle S. Borodulin K. Buman M. P. Cardon G. (2020). World Health Organization 2020 guidelines on physical activity and sedentary behaviour. *Br. J. Sports Med.* 54 1451–1462. 10.1136/bjsports-2020-102955 33239350 PMC7719906

[B4] Choi K. W. Chen C. Y. Stein M. B. Klimentidis Y. C. Wang M. J. Koenen K. C. (2019). Assessment of bidirectional relationships between physical activity and depression among adults: a 2-Sample mendelian randomization study. *JAMA Psychiatry* 76 399–408. 10.1001/jamapsychiatry.2018.4175 30673066 PMC6450288

[B5] Correll C. U. Solmi M. Veronese N. Bortolato B. Rosson S. Santonastaso P. (2017). Prevalence, incidence and mortality from cardiovascular disease in patients with pooled and specific severe mental illness: a large-scale meta-analysis of 3,211,768 patients and 113,383,368 controls. *World Psychiatry* 16 163–180. 10.1002/wps.20420 28498599 PMC5428179

[B6] Dunn A. L. Trivedi M. H. Kampert J. B. Clark C. G. Chambliss H. O. (2005). Exercise treatment for depression: efficacy and dose response. *Am. J. Prevent. Med.* 28 1–8. 10.1016/j.amepre.2004.09.003 15626549

[B7] Eldridge S. M. Chan C. L. Campbell M. J. Bond C. M. Hopewell S. Thabane L. (2016). CONSORT 2010 statement: extension to randomised pilot and feasibility trials. *Pilot Feasibil. Stud.* 2:64. 10.1186/s40814-016-0105-8 27965879 PMC5154046

[B8] Gatchel J. R. Rabin J. S. Buckley R. F. Locascio J. J. Quiroz Y. T. Yang H.-S. (2019). Longitudinal association of depression symptoms with cognition and cortical amyloid among community-dwelling older adults. *JAMA Netw. Open* 2:e198964. 10.1001/jamanetworkopen.2019.8964 31397865 PMC6692684

[B9] GBD 2019 Diseases and Injuries Collaborators (2020). Global burden of 369 diseases and injuries in 204 countries and territories, 1990-2019: a systematic analysis for the Global Burden of Disease Study 2019. *Lancet* 396 1204–1222. 10.1016/s0140-6736(20)30925-9 33069326 PMC7567026

[B10] Georg Hsu L. K. Wan Y. M. Chang H. Summergrad P. Tsang B. Y. Chen H. (2008). Stigma of depression is more severe in Chinese Americans than Caucasian Americans. *Psychiatry* 71 210–218. 10.1521/psyc.2008.71.3.210 18834272

[B11] Ghegan M. D. Angelos P. C. Stonebraker A. C. Gillespie M. B. (2006). Laboratory versus portable sleep studies: a meta-analysis. *Laryngoscope* 116 859–864. 10.1097/01.mlg.0000214866.32050.2e 16735890

[B12] Gorman J. M. (1996). Comorbid depression and anxiety spectrum disorders. *Depress Anxiety* 4 160–168. 10.1002/(sici)1520-639419964:4<160::Aid-da2<3.0.Co;2-j 9166648

[B13] Gormley S. E. Swain D. P. High R. Spina R. J. Dowling E. A. Kotipalli U. S. (2008). Effect of intensity of aerobic training on VO2max. *Med. Sci. Sports Exerc.* 40 1336–1343. 10.1249/MSS.0b013e31816c4839 18580415

[B14] Hallgren M. Kandola A. Stubbs B. Nguyen T.-T.-D. Wallin P. Andersson G. (2020). Associations of exercise frequency and cardiorespiratory fitness with symptoms of depression and anxiety - a cross-sectional study of 36,595 adults. *Mental Health Phys. Activity* 19:100351. 10.1016/j.mhpa.2020.100351

[B15] Hanson N. J. Scheadler C. M. Lee T. L. Neuenfeldt N. C. Michael T. J. Miller M. G. (2016). Modality determines VO2max achieved in self-paced exercise tests: validation with the Bruce protocol. *Eur. J. Appl. Physiol.* 116 1313–1319. 10.1007/s00421-016-3384-0 27150353

[B16] He X. Y. Li C. Qian J. Cui H. S. Wu W. Y. (2010). Reliability and validity of a generalized anxiety scale in general hospital outpatients. *Shanghai Arch. Psychiatry* 22 200–203. 10.3969/j.issn.1002-0829.2010.04.002

[B17] Josefsson T. Lindwall M. Archer T. (2014). Physical exercise intervention in depressive disorders: meta-analysis and systematic review. *Scandinavian J. Med. Sci. Sports* 24 259–272. 10.1111/sms.12050 23362828

[B18] Jurado-Fasoli L. Amaro-Gahete F. J. De-la-O A. Dote-Montero M. Gutiérrez A. Castillo M. J. (2018). Association between sleep quality and body composition in sedentary middle-aged adults. *Medicina (Kaunas, Lithuania)* 54:91. 10.3390/medicina54050091 30463242 PMC6262283

[B19] Kaseva K. Dobewall H. Yang X. Pulkki-Råback L. Lipsanen J. Hintsa T. (2019). Physical activity, sleep, and symptoms of depression in adults-testing for mediation. *Med. Sci. Sports Exerc.* 51 1162–1168. 10.1249/mss.0000000000001896 30694979

[B20] Kvam S. Kleppe C. L. Nordhus I. H. Hovland A. (2016). Exercise as a treatment for depression: a meta-analysis. *J. Affect. Disord.* 202 67–86. 10.1016/j.jad.2016.03.063 27253219

[B21] Leung C. M. Wing Y. K. Kwong P. K. Lo A. Shum K. (1999). Validation of the Chinese-Cantonese version of the hospital anxiety and depression scale and comparison with the Hamilton Rating Scale of Depression. *Acta Psychiatr Scand.* 100 456–461. 10.1111/j.1600-0447.1999.tb10897.x 10626925

[B22] Li H. Zheng D. Li Z. Wu Z. Feng W. Cao X. (2019). Association of depressive symptoms with incident cardiovascular diseases in middle-aged and older Chinese adults. *JAMA Netw. Open* 2:e1916591. 10.1001/jamanetworkopen.2019.16591 31800066 PMC6902756

[B23] Meng R. Yu C. Liu N. He M. Lv J. Guo Y. (2020). Association of depression with all-cause and cardiovascular disease mortality among adults in China. *JAMA Netw. Open* 3:e1921043. 10.1001/jamanetworkopen.2019.21043 32049295 PMC7212017

[B24] Nebiker L. Lichtenstein E. Minghetti A. Zahner L. Gerber M. Faude O. (2018). Moderating effects of exercise duration and intensity in neuromuscular vs. endurance exercise interventions for the treatment of depression: a meta-analytical review. *Front. Psychiatry* 9:305. 10.3389/fpsyt.2018.00305 30072923 PMC6060256

[B25] Neckelmann D. Mykletun A. Dahl A. A. (2007). Chronic insomnia as a risk factor for developing anxiety and depression. *Sleep* 30 873–880. 10.1093/sleep/30.7.873 17682658 PMC1978360

[B26] Nutt D. Wilson S. Paterson L. (2008). Sleep disorders as core symptoms of depression. *Dialogues Clin. Neurosci.* 10 329–336. 10.31887/DCNS.2008.10.3/dnutt 18979946 PMC3181883

[B27] O’Donovan G. Lee I.-M. Hamer M. Stamatakis E. (2017). Association of “Weekend Warrior” and other leisure time physical activity patterns with risks for all-cause, cardiovascular disease, and cancer mortality. *JAMA Int. Med.* 177 335–342. 10.1001/jamainternmed.2016.8014 28097313

[B28] Ohayon M. M. Roth T. (2003). Place of chronic insomnia in the course of depressive and anxiety disorders. *J. Psychiatric Res.* 37 9–15. 10.1016/S0022-3956(02)00052-3 12482465

[B29] Papasavvas T. Bonow R. O. Alhashemi M. Micklewright D. (2016). Depression symptom severity and cardiorespiratory fitness in healthy and depressed adults: a systematic review and meta-analysis. *Sports Med.* 46 219–230. 10.1007/s40279-015-0409-5 26446894

[B30] Passos G. S. Poyares D. Santana M. G. Garbuio S. A. Tufik S. Mello M. T. (2010). Effect of acute physical exercise on patients with chronic primary insomnia. *J. Clin. Sleep Med.* 6 270–275. 20572421 PMC2883039

[B31] Pate R. R. Pratt M. Blair S. N. Haskell W. L. Macera C. A. Bouchard C. (1995). Physical activity and public health. A recommendation from the Centers for Disease Control and Prevention and the American College of Sports Medicine. *JAMA* 273 402–407. 10.1001/jama.273.5.402 7823386

[B32] Pearson N. J. Johnson L. L. Nahin R. L. (2006). Insomnia, trouble sleeping, and complementary and alternative medicine: analysis of the 2002 national health interview survey data. *Arch. Internal Med.* 166 1775–1782. 10.1001/archinte.166.16.1775 16983058

[B33] Plasqui G. Westerterp K. R. (2007). Physical activity assessment with accelerometers: an evaluation against doubly labeled water. *Obesity (Silver Spring)* 15 2371–2379. 10.1038/oby.2007.281 17925461

[B34] Powell K. E. Paluch A. E. Blair S. N. (2011). Physical activity for health: what kind? How much? How intense? On top of what? *Annu. Rev. Public Health* 32 349–365. 10.1146/annurev-publhealth-031210-101151 21128761

[B35] Rogers A. E. Caruso C. C. Aldrich M. S. (1993). Reliability of sleep diaries for assessment of sleep/wake patterns. *Nurs. Res.* 42 368–372. 8247821

[B36] Schubert C. R. Cruickshanks K. J. Dalton D. S. Klein B. E. Klein R. Nondahl D. M. (2002). Prevalence of sleep problems and quality of life in an older population. *Sleep* 25 889–893. 12489896

[B37] Schuch F. B. Vancampfort D. Richards J. Rosenbaum S. Ward P. B. Stubbs B. (2016). Exercise as a treatment for depression: a meta-analysis adjusting for publication bias. *J. Psychiatric Res.* 77 42–51. 10.1016/j.jpsychires.2016.02.023 26978184

[B38] Spitzer R. L. Kroenke K. Williams J. B. W. Löwe B. (2006). A Brief Measure for assessing generalized anxiety disorder: the GAD-7. *Arch. Int. Med.* 166 1092–1097. 10.1001/archinte.166.10.1092 16717171

[B39] Tsai P. S. Wang S. Y. Wang M. Y. Su C. T. Yang T. T. Huang C. J. (2005). Psychometric evaluation of the Chinese version of the Pittsburgh Sleep Quality Index (CPSQI) in primary insomnia and control subjects. *Qual. Life Res.* 14 1943–1952. 10.1007/s11136-005-4346-x 16155782

[B40] Vancampfort D. Stubbs B. Smith L. Hallgren M. Firth J. Herring M. P. (2018). Physical activity and sleep problems in 38 low- and middle-income countries. *Sleep Med.* 48 140–147. 10.1016/j.sleep.2018.04.013 29908426

[B41] Yu D. S. (2010). Insomnia severity index: psychometric properties with Chinese community-dwelling older people. *J. Adv. Nurs.* 66 2350–2359. 10.1111/j.1365-2648.2010.05394.x 20722803

[B42] Yu M. Zhang X. Lu F. Fang L. (2015). Depression and risk for diabetes: a meta-analysis. *Canadian J. Diabetes* 39 266–272. 10.1016/j.jcjd.2014.11.006 25773933

[B43] Yu X. Tam W. W. Wong P. T. Lam T. H. Stewart S. M. (2012). The Patient Health Questionnaire-9 for measuring depressive symptoms among the general population in Hong Kong. *Compr. Psychiatry* 53 95–102. 10.1016/j.comppsych.2010.11.002 21193179

[B44] Zenebe Y. Akele B. W/Selassie M. Necho M. (2021). Prevalence and determinants of depression among old age: a systematic review and meta-analysis. *Ann. Gen. Psychiatry* 20:55. 10.1186/s12991-021-00375-x 34922595 PMC8684627

